# Hospital Progress in 1907

**Published:** 1908-01-04

**Authors:** 


### HOSPITAL ADMINISTRATION.

### CONSTRUCTION AND ECONOMICS.

### HOSPITAL PROGRESS IN 1907.

**King Edward's Hospital Fund.**

This Fund has made surprising progress during the year, and it is estimated its total receipts will not fall far short, if at all, of £480,000. Of these the general receipts, including the income from investments, which now exceeds £50,000 a year, will probably amount to about £170,000. Legacies and contributions to be retained as capital, including upwards of £200,000 from the estate of Samuel Lewis, deceased, exceed £312,000. Nearly £13,000 has been received from the estate of the late J. G. Lambert. The total investments of the Fund at the end of the year will probably amount to one and a half millions. These are splendid figures, and emphasise one of the most remarkable successes in the field of charity which has ever been initiated, and with which the Royal Family has been actively identified. The truth is the administration of the Fund has been sound. At no time in English history have the individual members of the Royal Family, from the King downwards, been as popular as they are to-day, and the policy pursued under the direct initiative of H.R.H. the Prince of Wales has won the confidence of all classes of people. It is not a little interesting to go back ten years and notice the criticisms and prophecies of non-success which were so persistently voiced by pessimists whose voices are still heard in connection with every movement of a progressive kind. No one living has probably witnessed a more delightful demonstration of the utter undoing of this type of individual than the King's Fund has afforded by its complete success. That success is now endorsed by both Houses of Parliament, and the future of the Fund must be intimately connected with the progress and development of our modern hospital system.

**The Central Hospital Council.**

The Central Hospital Council has made some progress during the year. It discharged a useful function by securing a substantial reduction in the water rate charged to metropolitan hospitals. To Mr. Charles Burt, the late Chairman of the Royal Free Hospital, belongs the just credit of this success, which procured a reduction of the cost of water to nearly every metropolitan hospital, and represents collectively a saving of some £5,000 a year in the working expenses of ninety-four such establishments. One useful result of the information published by the Central Hospital Council in this connection was to show precisely where intelligence had and where it had not been brought to bear on the administration and expenditure of particular hospitals. The figures eloquently testify to the wisdom of those institutions which have set up economy committees to examine and test every item of expenditure, so as to secure that no money shall be spent except upon evidence that in every case the best possible article will be supplied at the lowest possible cost. Every voluntary hospital, which has any ambition to be regarded as economically administered in the present day, must have an energetic economy committee as one of its most active and intelligent safeguards.

The Central Hospital Council has recently issued a draft of a bill to provide for the establishment of an official directory of nurses. The Act empowers the Privy Council to establish such a directory, and to provide for the appointment of an official registrar, with such clerks and servants as may be necessary to carry on the work. Power is taken to authorise the Privy Council to issue a provisional order regulating the particulars to be entered in the directory, and the conditions under which it shall be published, together with the fees to be charged for registration. It is further proposed that this order shall provide for the payment of an advisory committee to act as assessors for the purpose of enabling the registrar to decide any questions which may arise. The order is to prescribe the penalties or punishments attaching to any false and fraudulent declaration or certificate. Any excess in the expenditure rendered necessary by the passing of the Act, beyond the fees obtainable from registration, is to be paid by the Treasury. This bill has been submitted for the consideration of the principal metropolitan hospitals, but the result of this reference has not yet been reported.

**St. George's Hospital.**

An important new departure has been made at St. George's Hospital, where the office of lay superintendent has been abolished in favour of the appointment of a medical superintendent. We have frequently advocated this change in connection with the larger hospitals having medical schools. It would not be difficult, in our opinion, to prove on the facts that the administration of a large general hospital by a medical superintendent of capacity is not only economically sound in principle, but that in practice it tends to promote the efficiency of every department and the comfort and well-being of everybody concerned. Those who have lived in a large hospital where there is no medical superintendent are aware of the many defects, including slackness of discipline, and the many small but important discomforts and inefficiencies which must necessarily exist in any big establishment where there is no controlling mind in charge of the whole administration. In those great hospitals where the residents change every six months it is in our judgment not right to attempt to carry on the work without the constant supervision of a highly qualified and capable member of the medical profession as superintendent of the entire establishment. It is satisfactory to know that this opinion is being more and more held by all hospital authorities of any experience in this country. We, therefore, congratulate St. George's Hospital on the change which has recently taken place, and we hope that care will be taken to give the new superintendent ample authority, and that he may prove when appointed a highly capable and efficient officer. Should this prove to be the case, the cost per bed at St. George's Hospital is likely to show a considerable reduction in the near future.

### Hampstead General Hospital.

We dealt pretty fully during the year with the questions which have excited so much controversy in connection with this hospital. Put in a nutshell they amount to this. Is it right to erect a large general hospital containing more beds than the immediate requirements of the poor of the district in which it is situated warrant, to staff that hospital with none but local practitioners, and to expect the public at large to come forward and supply a large share of the income? In the present case, if the people of Hampstead and the local members of the profession there were prepared to supply all the revenue required, and to confine the admission of patients to local cases, being fit objects of charity, the institution would be able to escape much criticism. It would not in such circumstances be a public hospital in the wider meaning of the words, and might be left as a local institution to fulfil the local requirements in the way which best meets the prejudices and views of the locality in which it is situated. In fact, however, the Hampstead General Hospital has become one of the public hospitals of the metropolis, and the King's Fund has very properly laid it down that if it is to receive substantial aid from that Fund it must be equipped and managed in every department in such a way as to make it fulfil the conditions which now apply to a modern hospital. After a somewhat acrimonious and prolonged discussion the Governors of this institution have, by a small majority (195 votes against 187) decided to accept in principle the conditions of the King's Fund. We presume that the Hospital Authorities will now take steps to bring themselves into line with the requirements of the King's Fund, and that it may be found possible to finance the institution, many of the buildings of which have in our opinion been erected long before the financial position justified such a step. We beg to congratulate the chairman and the committee of management upon the courageous line they took in support of the principles which they hold to be essential, if this hospital is ever to fulfil its mission to the utmost extent possible.

